# Golgi Alpha1,2-Mannosidase IA Promotes Efficient Endoplasmic Reticulum-Associated Degradation of NKCC2

**DOI:** 10.3390/cells11010101

**Published:** 2021-12-29

**Authors:** Sylvie Demaretz, Elie Seaayfan, Dalal Bakhos-Douaihy, Nadia Frachon, Martin Kömhoff, Kamel Laghmani

**Affiliations:** 1 Centre de Recherche des Cordeliers, Sorbonne Université, Inserm, Université de Paris, F-75006 Paris, France; sylvie.demaretz@sorbonne-universite.fr (S.D.); elie.seaayfan@uni-marburg.de (E.S.); dalal.bakhos_aldouaihy@sorbonne-universite.fr (D.B.-D.); nadia.frachon@sorbonne-universite.fr (N.F.); 2 CNRS, ERL8228, F-75006 Paris, France; 3 Division of Pediatric Nephrology and Transplantation, University Children’s Hospital, Philipps-University, 35043 Marburg, Germany; Martin.Koemhoff@uk-gm.de

**Keywords:** kidney, NKCC2, protein quality control, ERAD, Golgi, alpha1,2-mannosidase IA, membrane, trafficking, Bartter syndrome, hypertension

## Abstract

Mutations in the apically located kidney Na-K-2Cl cotransporter NKCC2 cause type I Bartter syndrome, a life-threatening kidney disorder. We previously showed that transport from the ER represents the limiting phase in NKCC2 journey to the cell surface. Yet very little is known about the ER quality control components specific to NKCC2 and its disease-causing mutants. Here, we report the identification of Golgi alpha1, 2-mannosidase IA (ManIA) as a novel binding partner of the immature form of NKCC2. ManIA interaction with NKCC2 takes place mainly at the cis-Golgi network. ManIA coexpression decreased total NKCC2 protein abundance whereas ManIA knock-down produced the opposite effect. Importantly, ManIA coexpression had a more profound effect on NKCC2 folding mutants. Cycloheximide chase assay showed that in cells overexpressing ManIA, NKCC2 stability and maturation are heavily hampered. Deleting the cytoplasmic region of ManIA attenuated its interaction with NKCC2 and inhibited its effect on the maturation of the cotransporter. ManIA-induced reductions in NKCC2 expression were offset by the proteasome inhibitor MG132. Likewise, kifunensine treatment greatly reduced ManIA effect, strongly suggesting that mannose trimming is involved in the enhanced ERAD of the cotransporter. Moreover, depriving ManIA of its catalytic domain fully abolished its effect on NKCC2. In summary, our data demonstrate the presence of a ManIA-mediated ERAD pathway in renal cells promoting retention and degradation of misfolded NKCC2 proteins. They suggest a model whereby Golgi ManIA contributes to ERAD of NKCC2, by promoting the retention, recycling, and ERAD of misfolded proteins that initially escape protein quality control surveillance within the ER.

## 1. Introduction

The sodium balance and its regulation by the kidney, by regulating the extra-cellular volume, is a key determinant of long-term blood pressure (BP) control, as illustrated by rare monogenic syndromes affecting renal salt handling and considerably altering BP [1,2]. Indeed, although several factors contribute to the pathogenesis and maintenance of blood pressure elevation, renal mechanisms are believed to play a primary role, as hypothesized initially by Guyton [1]. The thick ascending limb of the loop of Henle (TAL) of the kidney is responsible for reabsorbing 20–30% of the filtered load of NaCl [3,4,5]. At the molecular level, TAL Na-Cl reabsorption is mediated by luminal Na-K-2Cl cotransporter NKCC2 [5]. As a consequence, NKCC2 transport function has a considerable impact on final urinary salt excretion, subsequently influencing long-term blood sodium balance [3,5]. Accordingly, inherited variations of the cotransporter and/or its regulators affect BP in humans [3,6,7,8]. Indeed, inactivation of NKCC2 causes type I Bartter syndrome (BS1), a life-threatening renal disease featuring low BP along with electrolyte abnormalities [9], whereas enhanced activity of the cotransporter has been linked to high BP [2,10,11,12,13]. Moreover, in several animal models of salt-sensitive hypertension [14,15], NKCC2 protein expression is increased and contributed to the development of hypertension. In addition to its role in the BP homeostasis, NKCC2 activity is also essential for the regulation of water balance [5,16]. In support of this notion, NKCC2 inactivation in patients with BS1 and BS5 causes development of severe polyuria [7,8,17]. Moreover, the impaired urinary-concentrating ability of aging kidneys is attributed, at least in part, to the reduced level of NKCC2 protein [18,19]. It is worth emphasizing that in all animal models of hypertension and aging, NKCC2 appears to be regulated mainly by post-translational mechanisms [5,16,19], underlining therefore the need to study the factors regulating the biogenesis and trafficking of NKCC2. Despite this, our knowledge of the molecular mechanisms underlying intracellular trafficking of wild-type and mutated NKCC2 proteins in mammalian cells, in particular its regulation by protein–protein interaction, remained very poor. Furthermore, the vast majority of previous reports focused mainly on the post-Golgi regulation of the cotransporter, in particular by phosphorylation [20,21,22], and therefore very little is known today about the regulation of the transporters at the ER and pre-Golgi level.

To operate correctly, NKCC2 must be properly targeted to the apical cell surface and expressed at a sufficient level to permit TAL cells to adapt appropriately to physiological or pathological challenges [3,23]. Like all transmembrane proteins, the preparation for NKCC2 appropriate trafficking to the cell membrane begins as the cotransporter protein is synthetized in the endoplasmic reticulum (ER) and continues as the protein transits through the Golgi network [24,25]. Likewise, similar to virtually all proteins destined for the plasma membrane, NKCC2 proteins translocated into the endoplasmic reticulum (ER) undergo quality control, such that only folded proteins are moved through the secretory pathway [26,27]. Control of protein folding in the ER is often related to N-glycosylation, a posttranslational modification initiated by the covalent linkage of a specific oligosaccharide (Glc_3_Man_9_GlcNAc_2_) to a nascent protein [28,29]. Once this oligosaccharide is transferred, several successive steps of protein maturation follow along the secretory pathway [28,30]. After removal of three glucose residues and the beginning of mannose trimming in the ER, as part of the quality control process, correctly folded proteins are transferred to the Golgi apparatus for maturation [26,28]. High mannose *N*-glycans are then further trimmed by specific mannosidases such as Golgi α1,2-mannosidases in the cis-Golgi [31,32]. Addition of GlcNAc, galactose, sialic acid and fucose sugars generate hybrid and complex *N-*glycans within the *medial-* and *trans-*Golgi compartments [33,34]. Subsequently, mature *N*-glycosylated proteins are targeted to their final destination. When the folding process fails, the terminal mannose residues from the core glycan structure are progressively trimmed, and defective proteins are translocated across the membrane for cytosolic proteasome degradation through mechanisms known as ERAD (endoplasmic reticulum-associated degradation [26,35]). The remaining aberrant proteins, which can form aggregates or cannot be detected by ERAD chaperones, are delivered to lysosomes for clearance via pathways collectively referred to as ER-phagy [36,37]. Nevertheless, some misfolded proteins can still escape the ER and advance to the Golgi, where they are subjected to a Golgi quality control (GQC) and targeted to lysosome or proteasome for degradation [38]. Both wild-type and mutant variants of transmembrane proteins are prone to ER quality control and degradation. The chloride channel protein CFTR (cystic fibrosis transmembrane conductance regulator), characterized as the first integral membrane mammalian ERAD substrate, is the best example [39,40]. Under normal conditions, up to 70% of wild-type CFTR is degraded by ERAD [39,40]. Even more impressively, deletion of phenylalanine 508 (ΔF508) the mutation of CFTR responsible for cystic fibrosis, reduces the folding efficiency, resulting in nearly 99% of mutant CFTR being degraded before it can reach the plasma membrane [39,40]. Similar to CFTR and several other transmembrane proteins such as HERG [41], ROMK [42] and NCC [43], we have previously shown that the majority of newly synthesized NKCC2 proteins were trapped in the ER and destined for degradation, a process that involves mainly the proteasome pathway [44,45,46]. ERAD begins with the detection of a misfolded protein by molecular chaperones [47]. The type of chaperones engaged in this process depend principally on the position of the folding lesion of the substrate which can occur either in the ER lumen, ER membrane, or cytoplasm [27,47]. Consequently, three different ERAD pathways have been proposed as ERAD-L (ERAD of substrates with misfolded lesions within the ER lumen), ERAD-M (membrane), and ERAD-C (cytoplasm) [27,47,48]. For instance, the ERAD of nascent CFTR involves both cytoplasmic and ER luminal chaperones [49]. Likewise, NCC, another transmembrane protein, engages several cytoplasmic chaperones for its ERAD [43,50]. Similar to the related kidney-specific electroneutral NCC, NKCC2 possesses 12 transmembrane regions, two large cytoplasmic domains, and a large ER-exposed exofacial loop [5]. Since NKCC2 contains domains in the ER and cytoplasm, an interaction of the cotransporter with ER molecular chaperones on either side or on both sides of the ER membrane, is conceivable. Moreover, given that the C-terminal domain of NKCC2 is the predominant cytoplasmic region [5], it is likely to be the major site of protein–protein interaction and therefore to play a paramount role in the biogenesis and trafficking of the cotransporter. In agreement with this notion, we previously identified several binding partners of NKCC2 C-terminus [46,51,52,53]. Among those, we showed that aldolase B and SCAMP2 bind to NKCC2 C-terminus at the post-Golgi level and regulate the subcellular redistribution of the cotransporter [51,52]. More recently, we provided evidence that STCH, Hsp70 and the protein-lectin OS9 interact with the immature form of NKCC2 mainly at the ER to regulate its ER-associated degradation [46,53]. In this report, we describe a novel protein–protein interaction between the C-terminal tail of NKCC2 and Golgi α1,2-mannosidase IA (Golgi ManIA, also called Man9-mannosidase), a ubiquitous protein that belongs to the family of class I α-1,2 mannosidases which includes the ER α-mannosidase I (MAN1B1) and two other Golgi α1,2-mannosidases,Golgi α-mannosidase IB [MAN1A2], and Golgi α-mannosidase IC [MAN1C1] [31,54,55,56]. Interestingly, an increasing body of evidence indicated that α-1,2-mannosidases are not only implicated in protein folding and maturation but also play a pivotal role in misfolded protein degradation [57,58,59,60]. Here, we show that ManIA interacts with the immature form of NKCC2 mainly at the cis-Golgi network and promotes its degradation by the proteasome pathway, revealing therefore an additional checkpoint in the surveillance and removal of misfolded NKCC2 proteins. Consequently, these findings may open up new avenues in studying the ER and Golgi quality control of NKCC2 proteins in order to help in the development of new strategies to prevent and/or treat kidney disorders related to aberrant NKCC2 trafficking and expression.

## 2. Materials and Methods

### 2.1. Yeast Two-Hybrid Assay

Yeast two-hybrid (Y2H) screening was performed as described previously in detail [51], using as bait the proximal region of the NKCC2 C terminus (residues 661–1095). Briefly, AH109 expressing the bait was mated with the Y187 yeast strain pretransformed with a human kidney cDNA library. For the selection of positive clones encoding putative interacting proteins, mated yeast cells were first grown on low stringency selection plates (−Leu, −Trp, −His) and then on high stringency selection plates (−Leu, −Trp, −His, −Ade). Selected colonies were also tested for β-galactosidase activity, and DNA from the positive clones was isolated from yeast cells using the RPM yeast plasmid isolation kit (BIO 101 Systems). After rescuing the prey plasmids by transformation into DH5 α bacteria (Invitrogen) and isolation using a Qiagen kit, cDNA plasmids were sequenced and assessed using the BLAST program.

### 2.2. Plasmid Constructions and Site Directed Mutagenesis

Generation of WT Myc-NKCC2, unglycosylated Myc-NKCC2 ((N442Q/N452Q) and WT EGFP-NKCC2 was previously described [45,46,51]. The mouse ManIA coding sequence was subcloned into the mammalian expression pcDNA3.1/V5 vector (Invitrogen, Paris, France) to generate WT ManIA-V5 construct. The plasmid comprising ManIA lacking its C-terminus tail (ManIA-ΔCter) was generated through amplification from the WT MAnIA-V5 expression construct using forward primer 5′CCGGAGCGATGAAGTTCGTGCTGCTGC 3′ and reverse primer 5′TTTCTCTTTGCCATCAATTTC 3′. The construct containing ManIA devoid from its *N*-terminus region (ManIA-ΔNter) was generated also from WT MAnIA-V5 plasmid by QuikChange site-directed mutagenesis method (Stratagene, Les Ulis, France) using forward primer 5′ GAGGGCAAAGATCAAAGAGTAGATGACCCATGCTTGGAAT 3′ and reverse primer 5′ ATTCCAAGCATGGGTCATCTACTCTTTGATCTTTGCCCTC 3′. All mutations and truncations were confirmed by sequencing.

### 2.3. Cell Culture

Opossum kidney cells (OKP cells) were maintained in DMEM (Gibco 42430) supplemented with 10% fetal bovine serum (Eurobio, Les Ulis, France), penicillin (100 U/mL), and streptomycin (100 U/mL) at 37 °C in a humidified atmosphere containing 5% CO_2_. Human embryonic kidney (HEK) 293 cells were grown in DMEM media complemented with 10% fetal bovine serum and 1% penicillin/streptomycin. For plasmid DNA transfection, cells were grown to 60–70% confluence before being transiently transfected for 5 h using Lipofectamine plus kit according to manufacturer’s instructions (Invitrogen, Paris, France). For protein degradation experiments, cells were treated with MG132 (2 μM) or chloroquine (100 μM) 6 h prior to cell lysis, as previously described [53,61]. Kifunensine (Sigma k1140, Saint-Quentin-Fallavier, France) was used at 25 µM 6 h before cell lysis.

### 2.4. Protein Preparation, Immunoblotting and Immunoprecipitation

After transfection, cells were washed with cold PBS before being solubilized in a lysis buffer containing 120 mM Tris/Hepes, pH 7,4; 150 mM NaCl, 5 mM EDTA, 3 mM KCl; 1% (*v*/*v*) Triton X-100 and protease inhibitors (Complete Roche 1697498, Meylan, France). Samples were then harvested and centrifuged at 16,000 rpm for 15 min at 4 °C. For immunoprecipitation, cells were solubilized with lysis buffer containing 0.4 M NaCl; 1.5 mM MgCl_2_; 10 mM Hepes, pH 7.9; 5% (*v*/*v*) glycerol; 0.5% (*v*/*v*) Nonidet P-40) and protease inhibitors (Complete, Roche Diagnostics). Immunoprecipitation was carried out using anti-V5 (Invitrogen) or anti-ManIA (Sigma ) antibody, and affinity purification using protein G-agarose beads (Dynabeads, Invitrogen, Paris, France). After incubation with protein G-agarose beads for 1 h at room temperature, the immunocomplex was washed in PBS (Invitrogen). The protein samples were then boiled in loading buffer, run on gradient 7.5% or 10% or 15% SDS-polyacrylamide gels, probed with primary antibodies of interest and horseradish peroxidase-conjugated secondary antibody. Proteins were visualized by enhanced chemiluminescence detection (Thermo Fisher Scientific, Les Ulis, France) according to the manufacturer′s instructions.

### 2.5. Immunocytochemistry

Twenty-four–forty-eight h post-transfection, confluent cells were washed with PBS^++^ (pH 8, 1 mM MgCl_2_, and 0.1 mM CaCl_2_). Cells were then fixed with 2% paraformaldehyde in PBS for 20 min at room temperature before being incubated with 50 mM NH_4_Cl, permeabilized with 0.1% Triton X-100 for 1 min. To block no specific antibody binding, cells were incubated with DAKO (antibody diluent with background-reducing components) for 30 min. Fixed cells were incubated for 1h at room temperature with the primary antibody of interest. The primary antibodies used in this study are the following: mouse anti-V5 (Invitrogen, Paris, France), mouse anti–Myc (Takara, Clontech, Saint-Germain-en-Laye, France), rat anti-V5 (Abcam, Paris, France), rabbit anti-Calnexin (Abcam), rabbit anti-Giantin (Abcam), rabbit anti-GM130 (Abcam), rabbit anti-ManIA (Abcam), The secondary antibodies used are the following: goat anti-rabbit Alexa Fluor 555 (Invitrogen), goat anti-rabbit Alexa Fluor 488 (Invitrogen), goat anti-rabbit FITC (DakoCytomation, Trappes, France), goat anti-mouse Texas red (Invitrogen), goat anti-rat Alexa Fluor 555 (Invitrogen), goat anti-rat Alexa Fluor 488 (Invitrogen), Alexa Texas Red conjugated anti-mouse (Jackson ImmunoResearch, Ely, UK), chicken anti-mouse Alexa Fluor 647 (Invitrogen). After incubations with primary and secondary antibodies of interest, cells were then washed with PBS and mounted with Vectashield.

### 2.6. Cycloheximide-Chase Assays

To monitor the stability and maturation of NKCC2, cycloheximide was added at a concentration of 100 μM to OKP or HEK cells 14−16 h post-transfection with NKCC2 plasmids. For the chase period, cell lysates were collected at 0, 1, 2, and 4 h after cycloheximide treatment and analyzed by immunoblotting.

### 2.7. siRNA Knockdown

ManIA siRNAs were purchased from Dharmacon as ON-TARGET plus SMART pools (L-012174-00-0005). HEK cells were first transfected with control or specific ManIA siRNAs with Lipofectamine RNAiMAX (Invitrogen) using the manufacturer′s specifications as performed previously [46,53]. One day after siRNA transfection, cells were transfected with NKCC2 plasmids. After 24–48 h, cell lysates were analyzed for each protein using the indicated antibodies.

### 2.8. Statistical Analyses

Data are expressed as mean ± SE. Differences between means were evaluated using paired or unpaired t test or ANOVA as appropriate. *p* < 0.05 was considered statistically significant.

## 3. Results

### 3.1. Identification of Golgi Mannosidase IA as a Novel NKCC2-Interacting Protein

**Figure 1:**
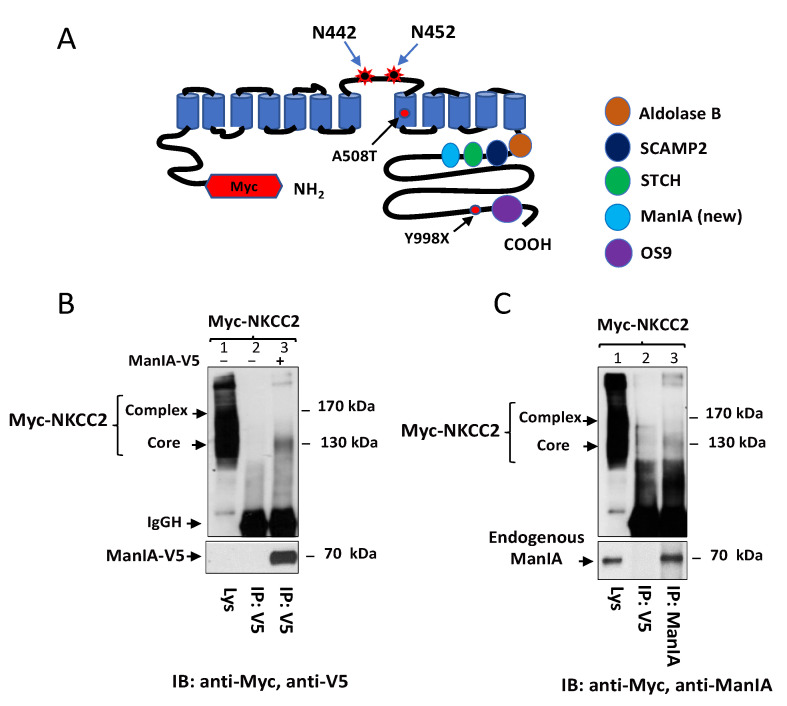
Identification of ManIA as a novel NKCC2-interacting protein. (**A**) A proposed topology for sodium-coupled chloride cotransporter NKCC2 N-terminally tagged with Myc. N442 and N452 are the potential *N*-glycosylation sites. NKCC2 mutations, A508T and Y998X, used in the present study are indicated by arrows and marked in rounds. We previously used mouse NKCC2 C terminus constructs as baits to screen a human kidney cDNA library using the yeast two-hybrid system. Similar to Aldolase B [51], SCAMP2 [52] and STCH [53], ManIA interact with the proximal region whereas OS9 binds to the distal region of NKCC2 C terminus [46]. (**B**) NKCC2 binds, in vivo, to ManIA in HEK cells. HEK cells transiently transfected with Myc-NKCC2 singly or in combination with ManIA-V5 were immunoprecipitated (IP) with anti-V5 anti-body (*lanes 2 and 3*). 5 % of total cell lysate (Lys) was resolved as positive control. Co-immunoprecipitated NKCC2 and ManIA proteins were detected by immunoblotting (IB) using anti-Myc and anti-V5 respectively (*lane 3*). IgGH, the heavy chain of IgG. The positions of immature (core glycosylated) and mature (complex-glycosylated) proteins of NKCC2 are indicated. The interaction of NKCC2 with ManIA implicates only the immature form of the cotransporter. (**C**) endogenous ManIA interacts with immature NKCC2. HEK cells overexpressing Myc-NKCC2 were immunoprecipitated (*IP*) with anti-V5 (negative control; *lane 2*), or anti-ManIA antibody from Sigma (*lane 3*). Co-immunoprecipitated NKCC2 (only the immature form) was detected by immunoblotting (*IB*) using anti-Myc antibody (*lane 3*).

As previously described [51], to identify novel NKCC2-interacting proteins, we used a yeast two-hybrid system to screen a human kidney cDNA expression library, using a series of bait fragments spanning the predicted cytoplasmic C-terminus (residues 661–1095) of murine NKCC2 named C1-term (residues 661–768), C2-term (residues 741–909), and C3-term (residues 898–1095). Using this strategy, we demonstrated that OS9 [46] interacts with the distal region of NKCC2 C-terminus, whereas STCH [53], SCAMP2 [52] and aldolase B [51] bind to the proximal region of this cytoplasmic tail of the cotransporter (Figure 1A). In the current work, we report the identification of a new binding partner of NKCC2 C-terminus. Indeed, among the positive clones of our yeast two-hybrid screening, one clone (number # 40) encoding full-length Golgi alpha1, 2-mannosidase IA (ManIA) was isolated as a candidate-binding protein of the proximal region (first 108 aa, residues 661–768) of NKCC2 C terminus. To check whether full-length NKCC2 might also interact with ManIA in a cellular context, Myc-NKCC2 construct was transiently expressed in the presence or absence of ManIA-V5 in HEK cells. Cell lysates were incubated with anti-V5 antibodies and the resultant immunoprecipitates were subjected to SDS-PAGE and Western blot analysis. As shown in Figure 1B, *lane 3,* NKCC2 protein was detected in ManIA immunoprecipitates witnessing interaction of the proteins. Importantly, the interaction engages only the immature and core-glycosylated forms of the cotransporter. Moreover, the association appears to be specific since Myc-NKCC2 protein was not recovered in control experiments (Figure 1B, *lane 2)* in which cells were transfected in the absence of ManIA-V5.

To discount the possibility that the interaction described above is simply due to aggregation and nonspecific binding of proteins because of heterologous expression systems, we next tested whether endogenous ManIA protein and NKCC2 interact in HEK cells. To address this question, we used a rabbit ManIA antibody raised against native ManIA protein. Importantly, western blot analysis, following immunoprecipitation with anti-ManIA antibody, detected a protein band around 70 kDa, corresponding to the expected size band of ManIA protein. Most importantly, immunoprecipitation of endogenous ManIA protein brought down only the immature form NKCC2, indicating therefore physical interaction between ManIA and the immature form of the cotransporter (Figure 1C, *lane 3*). Of note, Myc-NKCC2 protein was not detected in control experiments in which immunoprecipitations were carried out using mouse anti-V5 antibodies indicating, again, that the interaction is specific (Figure 1C, *lane 2*). Taken in concert, these findings clearly indicate that, similar to exogenously expressed ManIA, endogenous ManIA binds in vivo to NKCC2, an interaction that implicates only the immature form of the cotransporter.

### 3.2. NKCC2 and ManIA Co-Localize Mainly at the Cis-Golgi Compartment

**Figure 2:**
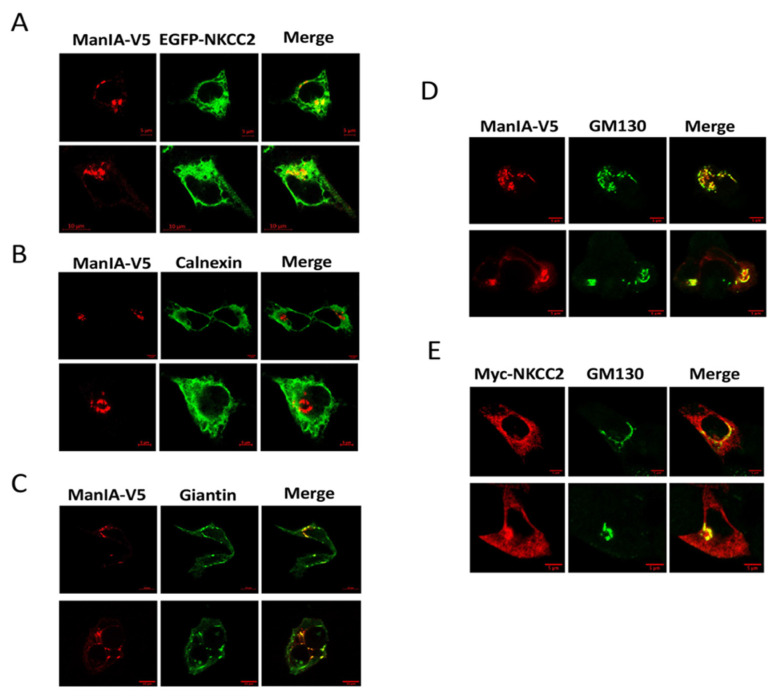
ManIA co-localizes with NKCC2 mainly at the cis-Golgi network. (**A**) imunofluorescence confocal microscopy showing distribution of EGFP-NKCC2 (green) and ManIA-V5 (red) in HEK cells. Fixed and permeabilized cells were stained with mouse anti-V5 for ManIA (Texas Red). The yellow color (merged image) indicates colocalization of the proteins. (**B**–**D**) comparison between the cellular localization of ManIA and ER or Golgi markers. Cells transfected with NKCC2 tagged with Myc and ManIA tagged with V5 were fixed after transfection, immunostained with anti -calnexin (ER marker) or Giantin (cis- and median Golgi marker) or GM130 (cis-Golgi marker) antibodies and visualized with texas-red of ManIA (red) and FITC conjugated secondary antibodies for each organelle marker. Analysis was performed by confocal laser scanning microscopy. (**E)** comparison between immmunostaining of Myc-NKCC2 with that of the cis-Golgi marker GM130. The yellow color (merged image) indicates colocalization of the proteins. Bars, 5 or 10 μm.

The association of ManIA with only the immature form of NKCC2 strongly suggests that the two proteins may interact at the ER. However, given that the N-glycan of glycoproteins at the entrance of the Golgi apparatus is still of high mannose type, it is also conceivable that NKCC2 interaction with ManIA may occur at the cis-Golgi network. To check whether ManIA interaction with NKCC2 takes place at the ER and/or the cis-Golgi, we visualized the subcellular distribution of NKCC2 and ManIA by immunofluorescence microscopy in renal cultured cells. To that end, HEK cells were first co-transfected with EGFP-NKCC2 and ManIA-V5 proteins. Of note, we have previously shown that, similar to Myc, N-terminal tagging of NKCC2 with EGFP does not alter intracellular trafficking of the cotransporter [45,51]. As shown in Figure 2A, NKCC2 (Green) co-localized with ManIA (red), indicating that these two proteins have overlapping subcellular localization. However, by contrast to the colocalization of NKCC2 with OS9 [46] and STCH [53], the two colocalized proteins did not display an immunofluorescence staining pattern that is more restricted to a perinuclear ER-like distribution. Indeed, ManIA appeared in a punctuate pattern distribution, which already strongly suggests that the interaction is very unlikely to take place at the ER. To confirm this, the subcellular localization of ManIA was compared with that of the ER marker calnexin. As can be seen in Figure 2B, ManIA did not colocalize with the ER marker calnexin (Figure 2B, middle left panels), arguing against the possibility of an interaction between NKCC2 and ManIA at the ER. To further analyze the subcellular distribution of ManIA, more colocalization experiments were performed using Golgi markers GM130 and giantin. As shown in Figure 2C (lower left panels), ManIA intensively colocalized with the cis/medial Golgi marker giantin clearly indicating that the enzyme is expressed in the Golgi network. Most importantly, ManIA (Figure 2D) and NKCC2 (Figure 2E) co-localized also with the cis-Golgi marker GM130 strongly suggesting that the main site of NKCC2 binding to ManIA is the cis-Golgi compartment in HEK cells.

### 3.3. ManIA Decreases NKCC2 Stability and Maturation

**Figure 3:**
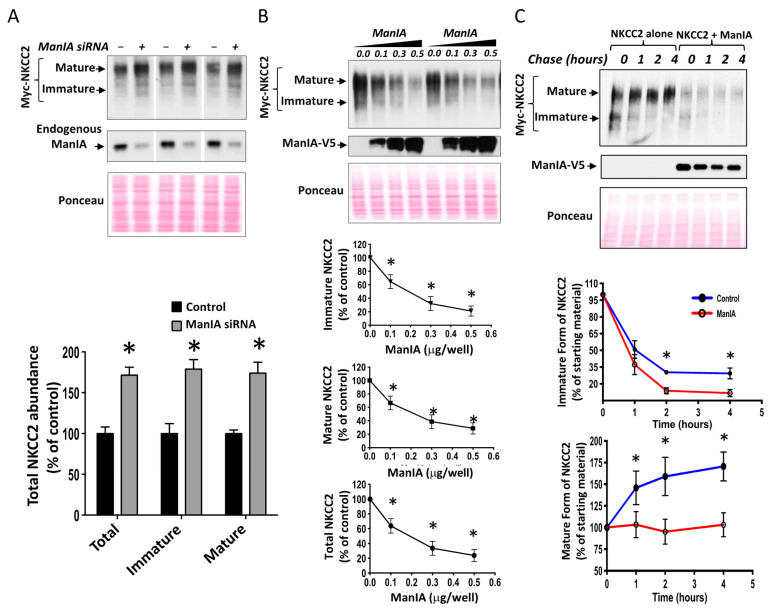
ManIA impedes NKCC2 stability and maturation. (**A**) Knockdown of endogenous ManIA in HEK cells increases immature and mature NKCC2 protein abundance. *Upper panels*, Representative immunoblot showing the effect of ManIA knockdown on NKCC2. HEK cells were transfected with NKCC2 without (−) or with specific ManIA siRNA (+). 48 h post-transfection, total cell extract from each sample was run on a parallel SDS gel and immunoblotted for total NKCC2 using Myc antibody. *Lower panels,* quantitative analysis of NKCC2 protein abundance. Data were normalized with the loading control (Ponceau staining) from the same lane in each membrane and are expressed as percentage of control. * *p* < 0.0004 (*n* = 3) versus control. (**B**) Total NKCC2 protein abundance is reduced by ManIA in a dose-dependent fashion. HEK cells were co-transfected with Myc-NKCC2 (0.1 μg/well) and increasing amounts of ManIA (0.1–0.5 μg/well) as indicated. NKCC2 proteins were detected by Western blotting with Myc antibody (*left panel*). *Lower panels*, densitometric analysis of total, immature and mature NKCC2 proteins with or without ManIA co-expression. Data are expressed as a percentage of control *±* SE, * *p* < 0.05 (*n* = 4), versus control. (**C**), ManIA decreases NKCC2 stability and maturation. *Upper panel*, representative immunoblot showing cycloheximide chase analysis of NKCC2 in the presence or absence of ManIA-V5. 14–16 h post-transfection, HEK cells transiently expressing WT NKCC2 alone or in combination with ManIA, were chased for the indicated time after addition of cycloheximide. Total cell lysates were separated by SDS-PAGE and probed by anti-Myc and anti-V5 antibodies. *Lower panels*: Quantitative analysis of NKCC2 stability and maturation. The density of the mature and immature form of NKCC2 proteins was normalized to the density at time 0. * *p* < 0.05 versus control (*n* = 4).

To determine the functional implications of ManIA-NKCC2 interaction, we first tested the impact of ManIA knock-down on total NKCC2 protein abundance using small interference RNA (siRNA). To this end, a sequential transfection in HEK cells was performed. Cells were first transfected with siRNAs for at least 24 h, before transfection with Myc-NKCC2 plasmids. As can be seen in Figure 3A, ManIA knockdown enhanced the abundance of the immature and mature forms of NKCC2, increasing therefore total cellular NKCC2 protein. Hence, these data strongly suggest that ManIA interacts with NKCC2 to impede the biogenesis of the cotransporter. To confirm the regulation of NKCC2 biogenesis by ManIA, we next investigated the effect of ManIA overexpression on NKCC2 in HEK cells. Towards this, Myc-NKCC2 cDNA was transfected into HEK cells in the absence (empty vector) or the presence of ManIA-V5. As illustrated in Figure 3B, ManIA co-expression strikingly decreased the amount of immature and mature forms of NKCC2 (Figure 3B) in a dose dependent fashion, which is in agreement with the increase of NKCC2 expression following ManIA knock-down. These data strongly suggest therefore that ManIA is indeed involved in the regulation of NKCC2 biogenesis.

The interaction of ManIA only with the immature form of NKCC2 and the downregulation of the latter are likely due to changes in NKCC2 protein stability and maturation. To test this hypothesis, we analyzed the effect of ManIA on the stability and maturation of NKCC2 using cycloheximide assay. Towards that end, 14 to 16 h after transfection of HEK cells with NKCC2 singly or in combination with ManIA, cells were incubated with cycloheximide for 0, 1, 2, and 4 h to block protein synthesis and immunoblotting was used to detect changes in NKCC2 protein maturation and degradation rate. During the chase period, under control conditions, the immature form of NKCC2 was progressively converted to a more slowly migrating band, representing the mature form. As shown in Figure 3C, ManIA co-expression induced a rapid decay of the immature NKCC2 protein during the chase period. Of note, the decrease in half-life of immature NKCC2 form represents its degradation, as well as its conversion to the mature form of the cotransporter. Importantly, ManIA co-expression heavily impaired maturation of glycosylated NKCC2 by strikingly impeding protein conversion from immature to mature form (Figure 3C, upper and lower panels), an effect that was clearly accentuated during the chase period (Figure 3C, lower right panel). Collectively, these data demonstrate that ManIA interacts with immature form of NKCC2 protein to promote efficient NKCC2 degradation impairing, therefore, maturation of the cotransporter in HEK cells.

### 3.4. ManIA Impairs NKCC2 Maturation Independently of the Expression System

**Figure 4:**
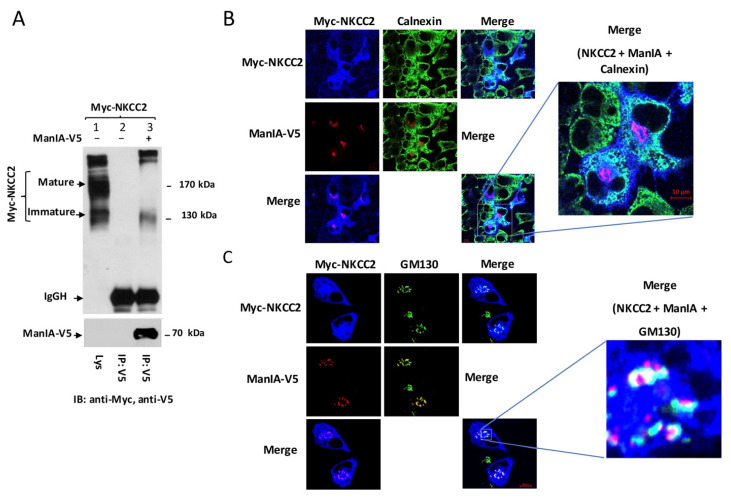
ManIA interacts with immature NKCC2 at the cis-Golgi in OKP cells, a second renal cell line. (**A**) ManIA interacts with immature NKCC2 in OKP cells. Cells were transiently transfected with Myc-NKCC2 either singly or in combination with ManIA-V5 construct. Cell lysates were immunoprecipitated (IP) with anti-V5 antibody. NKCC2 protein was recovered from anti-V5 immunoprecipitates mainly in its immature form (lane 3). (**B**,**C**) Comparison of the cellular localization of Myc-NKCC2 and ManIA-V5 with the ER marker calnexin or the cis-Golgi marker GM130. Fixed and permeabilized cells were stained with mouse anti-Myc, rat anti-V5 and rabbit anti-calnexin (**B**) or anti-GM130 (**C**) antibodies. The merge color indicates overlap between the Myc tag of NKCC2 protein (Alexa Fluor 647, Blue), the V5 tag of ManIA (Alexa Fluor 555 goat anti-rat, red) and the ER marker calnexin (FITC, green) or the cis-Golgi marker GM130 (FITC, green) and representing colocalization of the proteins. Bars, 5 μm.

Because protein subcellular distributions, ER quality control mechanism and protein–protein interaction phenomena may depend on the expression system used, we sought to confirm our observations by conducting the experiments in another renal cell line, OKP cells. As shown in Figure 4A, immunoprecipitation of ManIA followed by immunoblotting for NKCC2 revealed robust co-immunoprecipitation of the two proteins as illustrated by the intensity of the bands corresponding to NKCC2. Again, the interaction implicates only the immature NKCC2 despite the predominant expression of the complex-glycosylated and mature form of the cotransporter under these experimental conditions. (Figure 4A, *lane 3*). To verify whether, similar to HEK cells, the site of interaction between NKCC2 and ManIA is also the cis-Golgi network, the subcellular localization of the proteins was analyzed by confocal microscopy. As illustrated in Figure 4B, in cells co-expressing myc-NKCC2 and ManIA-V5, the two proteins colocalize (pink color). However, whereas in cells overexpressing ManIA, NKCC2 largely colocalized with calnexin, the staining of ManIA did not overlap with this ER marker (Figure 4B, middle panels), demonstrating again that their interaction does not occur at the ER. Most importantly, similar to HEK cells, ManIA largely colocalized with the cis-Golgi marker GM130 in OKP cells (Figure 4C). Likewise, some NKCC2 proteins appear to colocalize also with the cis-Golgi marker GM130 (Figure 4C). Accordingly, NKCC2 and ManIA displayed simultaneous colocalization with GM130 (Figure 4C, lower right panel, white color) providing therefore additional evidence that NKCC2 and ManIA interact and that this association occurs mainly at the cis-Golgi network.

**Figure 5:**
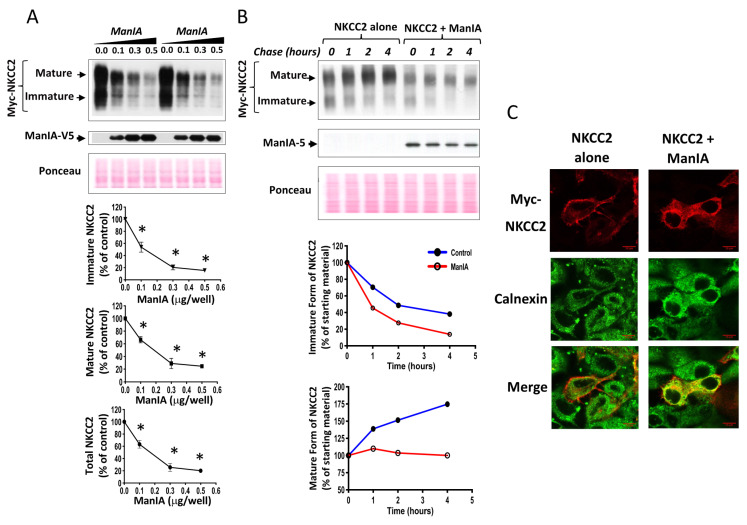
ManIA impairs NKCC2 maturation and stability independently of the expression system. (**A**) ManIA co-expression reduces Total NKCC2 protein abundance in a dose-dependent fashion. OKP cells were co-transfected with Myc-NKCC2 (0.1 μg/well) and increasing amounts of ManIA (0.1–0.5 μg/well) as indicated. NKCC2 proteins were detected by immunoblotting using Myc antibody (left panel). *Lower panels,* quantitation of steady state mature, immature, and total NKCC2 expression levels with or without ManIA co-expression. Data were normalized to the indicated loading control (Ponceau staining) from the same lane in each membrane and are expressed as percentage of control *±* SE, *, *p* < 0.04 (*n* = 3), versus control. (**B**) Analysis of NKCC2 stability and maturation monitored by cycloheximide-chase upon ManIA expression. OKP cells were co-transfected with NKCC2 together with a control vector or ManIA-V5 construct. 16 h later, cell lysates were prepared at the indicated time points after cycloheximide treatment (100 μM). Total protein extracts are subjected to SDS-PAGE and probed using anti-Myc and anti-V5 antibodies. The density of the mature and immature forms of NKCC2 proteins was normalized to the density at time 0. (**C**) Effect of ManIA on subcellular distribution of NKCC2. OKP cells transfected with Myc-NKCC2 alone or with ManIA-V5 were stained with mouse anti-Myc (Texas Red; red) and rabbit anti-calnexin (FITC; green). The yellow color in the merged image indicates colocalization of Myc-NKCC2 (red) with the ER marker (green).

To study the effect of ManIA on NKCC2 biogenesis in OKP cells, we checked again the effect of ManIA co-expression on the cotransporter protein levels. As show in Figure 5A, ManIA co-expression decreased again NKCC2 protein abundance in a dose-dependent fashion. To investigate whether this effect is related to the ERAD system, we next assessed the effect of ManIA on NKCC2 stability and maturation. As illustrated in Figure 5B, ManIA overexpression decreased the stability of immature NKCC2 protein which is in agreement with the data described above in HEK cells. Again, the decrease in immature NKCC2 stability in cells overexpressing ManIA was associated with a striking alteration in the conversion of the immature to the mature protein form of NKCC2. Taken together, these data demonstrate that, similar to HEK cells, ManIA targets the immature form of NKCC2 to the ER-associated degradation pathway in OKP cells, clearly indicating that ManIA effects on the cotransporter expression are independent of the expression system.

To further elucidate the consequence of ManIA effect on NKCC2 maturation, the subcellular localization of NKCC2 proteins in the presence and absence of ManIA co-expression was compared with the distribution of an ER marker, calnexin. In full agreement with our previous reports [13,45,46,52,61], when transfected alone, NKCC2 was essentially confined to the cell surface. Consequently, in the absence of ManIA overexpression, NKCC2 staining clearly surrounded the calnexin signal (Figure 5C, upper panel). Upon ManIA co-expression, NKCC2 surface expressed was greatly reduced and an intensive perinuclear pattern of staining was observed for NKCC2 with marked colocalization with the ER marker calnexin (Figure 5C, lower panel). These results strongly indicate that misfolded NKCC2 proteins, that initially escaped the ER, are captured by ManIA to be delivered back to the ER promoting therefore their retention. Collectively, our data provide evidence that ManIA co-expression promotes the ER retention and associated degradation of NKCC2, impairing therefore NKCC2 maturation and appropriate targeting to the cell surface expression.

### 3.5. ManIA Promotes NKCC2 ERAD in a Proteasome-Dependent Manner

**Figure 6:**
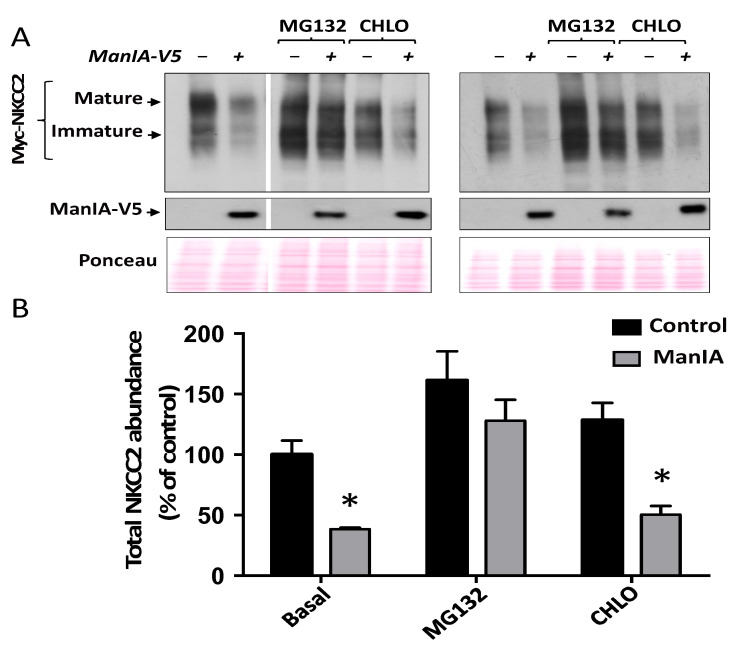
ManIA decreases NKCC2 expression in a proteasome-dependent manner. (**A)** Effect of MG132 and chloroquine on ManIA-induced down-regulation of NKCC2. 18 h post-transfection with NKCC2 and ManIA constructs, HEK cells were treated with or without 10 μM MG132 or 100 μM chloroquine for 6 h prior to cell lysis as previously [53,61].The cell lysates were subjected to immunoblotting with anti-Myc and anti-V5 antibodies. (**B**) Densitometric analysis of NKCC2 bands from untreated and treated cells with MG132 or chloroquine (CHLO). Data are expressed as percentage of control ±S.E. * *p* < 0.008 versus control (*n* = 3).

The experiments described above strongly suggest that enhanced expression of ManIA promotes ER retention and degradation of misfolded NKCC2 proteins. This mechanism implicates, in general, activation of the proteasome pathway and/or the lysosomal machinery [48,62,63,64,65]. Hence, we anticipated that the use of proteasome and lysosome inhibitors might help to elucidate the possible mechanisms of NKCC2 degradation upon ManIA co-expression. To check whether the proteasomal and/or lysosomal degradation pathways are involved, 16–18 h post-transfection cells were treated for 6 h with 2 μM MG132 or 100 μM chloroquine as previously described [53,61] and their lysates were subjected to Western blotting analysis. In agreement with our previous studies [46,53,61], MG132 treatment significantly increased total cellular NKCC2 protein abundance (Figure 6A,B). Most importantly, MG132 protected NKCC2 against ManIA-induced down-regulation of NKCC2 (Figure 6A,B), strongly suggesting that a proteasome-dependent mechanism underlies ManIA-mediated degradation of the cotransporter. In contrast to MG132, chloroquine was without effect on ManIA-induced down-regulation of NKCC2 (Figure 6A,B), clearly indicating that the lysosome pathway is not involved in the action of ManIA on the cotransporter. Altogether, these results strongly suggest that ManIA decreases NKCC2 expression by enhancing its ERAD in a proteasome-dependent manner.

### 3.6. Effect of Mannose Trimming of NKCC2 on the ManIA-Dependent ERAD Pathway

**Figure 7:**
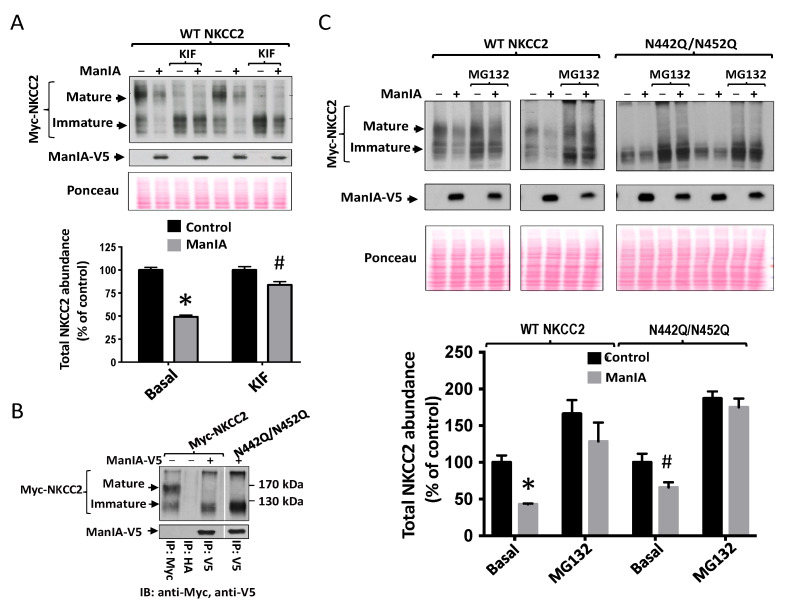
Role of mannose trimming in NKCC2 regulation by ManIA. (**A**) Effect the mannose trimming inhibitor kifunensine on ManIA-induced downregulation of NKCC2. 18 h post-transfection, OKP cells transiently transfected with Myc-NKCC2 alone or with ManIA-V5, were treated with 25 μM of kifunensine (KIF) or without for 6 h prior to cell lysis. The cell lysates were subjected to SDS-PAGE and immunoblotted with anti-Myc and anti-V5 antibodies. *Bottom*, densitometric analysis of NKCC2 bands from untreated and treated cells with kifunensine (KIF). Data are expressed as percentage of control ± SE. *, *p* < 0.001, #, *p* < 0.02 *versus* control (*n* = 4). (**B**) co-immunoprecipitation of ManIA with non-glycosylated NKCC2 (N442Q/N452Q). Cell lysates from cells transfected with WT NKCC2 or N442Q/N452Q in the presence or absence of ManIA-V5 were immunoprecipitated (*IP*) with anti-Myc or anti-V5 antibody. Similar to WT NKCC2, N442Q/N452Q protein co-immunoprecipitated with ManIA was detected by immunoblotting (*IB*) using anti-Myc (*lane 3*). (**C**) Effect of ManIA on non-glycosylated NKCC2. HEK Cells were transfected with Myc-NKCC2 or N442Q/N452Q in the presence or absence of ManIA-V5. 18 h post-transfection, cells were treated with or without 10 μM MG132 for 6 h prior to cell lysis as previously described [53,61]. Cell lysates were then subjected to immunoblotting analysis for Myc-NKCC2 and ManIA-V5. *Lower panel*, quantitation of steady-state total NKCC2 expression levels with or without ManIA co-expression. Data were normalized to the indicated loading control (Ponceau staining) from the same lane in each membrane and are expressed as percentage of control *, *p* < 0.004, #, *p* < 0.05 *versus* control (*n* = 3).

NKCC2 is N-glycosylated at two sites, N442 and N452, located in the long extracellular loop [5,51]. Consequently, since NKCC2 protein is modified by *N*-linked glycosylation [51] and given that several studies demonstrated that mannose trimming plays a crucial role in the ER-associated proteasomal degradation of glycoproteins [53,66,67], we next assessed the effect of the mannose trimming inhibitor kifunensine on ManIA-induced down-regulation of NKCC2. It is worth noting that kifunensine is commonly used to inhibit all α-mannosidases I (ER mannosidase I, Golgi mannosidases and EDEMs) that are implicated in the ERAD of glycoprotein [41,53,66,67,68]. To this end, 16–18 h post-transfection, cells were treated in the absence (control) or presence of kifunensine for 6 h. As expected, the inhibition of mannose trimming prevented the complex-glycosylation of NKCC2 resulting therefore in the loss of the mature form of NKCC2 with a lower band around 130 (immature form) kDa remaining (Figure 7A). Importantly, the presence of kifunensine greatly reduced ManIA-induced protein degradation of the cotransporter, which clearly indicates that mannose trimming is involved in ManIA effect on NKCC2 (Figure 7A). Of note, the persistence of a small but significant ManIA effect on NKCC2 in the presence of kifunensine suggests that, at least in part, the N-glycosylation of NKCC2 is not fully required for ManIA-induced protein degradation of the cotransporter.

To further study the role of the N-Glycan in ManIA-induced down-regulation of NKCC2, we next studied the role of N-glycosylation of NKCC2 on its interaction with ManIA. To this end, we assessed the effect of mutating the two predicted glycosylation sites, N442 and N452 of NKCC2, to glutamine (Q), on ManIA interaction with the cotransporter. As expected, elimination of the two sites of N-glycosylation; (N442Q/N452Q) resulted in the complete loss of NKCC2 glycosylation with a lower band around 130 kDa remaining (Figure 7B), corresponding to the unglycosylated form of the cotransporter. As can be seen in Figure 7B, ManIA interacts, again, only with the immature form of WT NKCC2. Importantly, despite the complete removal of N-Glycan from NKCC2, ManIA was still able to bind to the unglycosylated form of the cotransporter, clearly indicating therefore that ManIA binding to NKCC2 is not N-glycan dependent. These findings prompted us to compare the effect of ManIA on WT NKCC2 with that on *unglycosylated* NKCC2. As can be seen in Figure 7C, ManIA co-expression strikingly decreased wild-type (WT) NKCC2 protein expression, an effect that is prevented, once again, with the proteasome inhibitor MG132. Importantly, mutating the N-glycosylation sites of NKCC2 significantly reduced the effect of ManIA on the cotransporter expression strongly suggesting that N-glycosylation plays an important role in ManIA-induced down-regulation of NKCC2. Of note, the mutation of NKCC2 N-glycosylation sites did not fully inhibit ManIA effect on NKCC2, which is fully in agreement with the data obtained after kifunensine treatment. Importantly, the persistent effect of ManIA on un-glycosylated NKCC2 was completely abrogated by MG132, further corroborating the notion that ManIA promotes the proteasomal degradation of the cotransporter.

### 3.7. Role of the Cytoplasmic Domain of ManIA in its Effect on NKCC2

**Figure 8:**
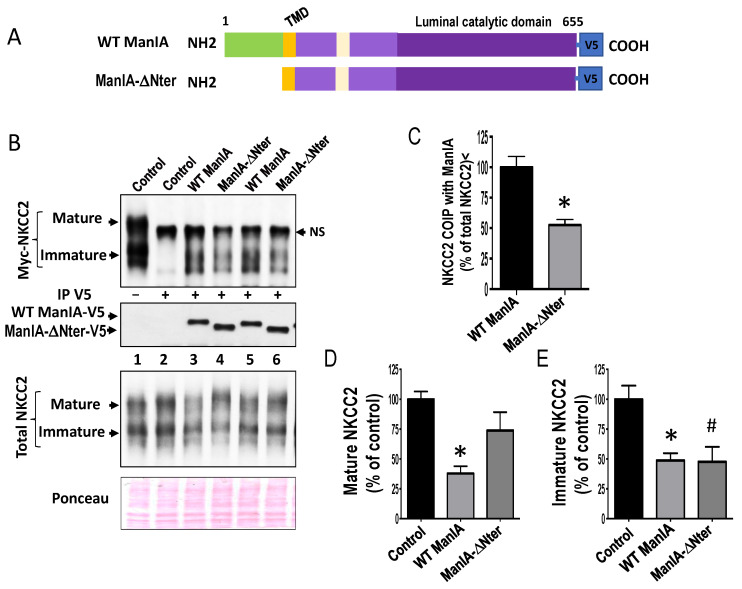
Role of the cytoplasmic domain of ManIA on its effect on NKCC2. (**A**) schematic of full-length of WT ManIA-V5 and the version of the protein truncated of its N-terminal cytoplasmic region (green). TMD, transmembrane domain. (**B**) Role of the cytoplasmic domain of ManIA on its interaction with NKCC2. HEK Cells were transiently transfected with Myc-NKCC2 either singly or in combination with WT ManIA-V5 or ManIA-ΔNter. Cell lysates were immunoprecipitated (IP) with anti-V5 antibody. Co-immunoprecipitated and total NKCC2 and ManIA proteins were detected by immunoblotting using anti-Myc and anti-V5 antibodies. NS, non-specific. (**C**) quantitation of co-immunoprecipitated NKCC2 protein with WT ManIA or ManIA-ΔNter normalized to total NKCC2 protein for each lane. (**D**,**E**), Effects of WT ManIA and ManIA-ΔNter on mature and immature NKCC2, respectively. densitometric analysis of mature (**D**) and immature (**E**) NKCC2 bands in the absence or presence of WT ManIA or ManIA-ΔNter. Data were normalized to the indicated loading control (Ponceau staining) from the same lane in each membrane and are expressed as percentage of control ± S.E. * *p* < 0.03, # *p* < 0.05 *versus* control (*n* = 4).

Mammalian Golgi 1,2-mannosidase IA is a type II transmembrane protein with an *N*-terminus on the cytoplasmic side and the C-terminus on the luminal side [55]. As mentioned above, using the yeast two-hybrid, we identified ManIA as a binding partner of the C-terminal and cytoplasmic side of NKCC2. Hence, it is very likely that ManIA interaction with the cotransporter involves, at least, the *N*-terminus and cytoplasmic side of this Golgi mannosidase. To verify this hypothesis, we studied the influence of deleting the *N*-terminus of ManIA on its interaction with NKCC2 using co-immunoprecipitation assay. To this end, cells were co-transfected with NKCC2 with full length ManIA (WT ManIA) or ManIA devoid of its *N*-terminus and cytoplasmic region (ManIA-ΔNter) (Figure 8A). Cell lysates were incubated with anti-V5 antibodies, and the resultant immunoprecipitates were fractioned by SDS page and probed for the presence of Myc-NKCC2. As can be seen in Figure 8B, immunoprecipitation of ManIA protein pulled down, again, only the immature form NKCC2. Most importantly, the ability of ManIA to interact with the immature NKCC2 protein was greatly reduced when deleted from its *N*-terminus (Figure 8B). Indeed, the abundance of the immature NKCC2 proteins that was recovered with ManIA-ΔNter immunoprecipitates was diminished when compared with WT ManIA (Figure 8C, *lanes 3 and 5* vs. *lanes 4 and 6*). Hence, these results strongly suggest that the cytoplasmic tail of ManIA is involved in its interaction with the cotransporter in HEK cells.

To assess the consequence of this decrease in the ability of ManIA-ΔNter to interact with NKCC2, we then compared the effect of ManIA-ΔNter on the expression of NKCC2 protein with that of WT ManIA. As shown in Figure 8B, overexpression of WT ManIA decreased again the total NKCC2 protein abundance. Interestingly, in cells co-expressing ManIA-ΔNter, although the decrease in NKCC2 protein abundance persisted, it seemed to be reduced when compared to WT ManIA (Figure 8B, *lanes 3 and 5* vs. *lanes 4 and 6*). Most importantly, overexpressing ManIA-ΔNter reversed the ManIA effect on the mature form of NKCC2 (Figure 8B, *lanes 3 and 5* vs. *lanes 4 and 6,* and Figure 8D), whereas it was without effect on the regulation of the immature form of the cotransporter (Figure 8B, *lanes 3 and 5* vs. *lanes 4 and 6,* and Figure 8E). Altogether, these data strongly suggest that the interaction of ManIA with NKCC2 involves, at least in part, the cytoplasmic region of this Golgi mannosidase. Moreover, they reveal that this interaction plays a crucial role in the retention of misfolded NKCC2 proteins. Consequently, they further support the notion that the decrease in NKCC2 protein expression and maturation was, at least in part, a result of a protein-protein interaction involving ManIA.

### 3.8. Role of the Catalytic Domain of ManIA on its Effect on NKCC2

**Figure 9:**
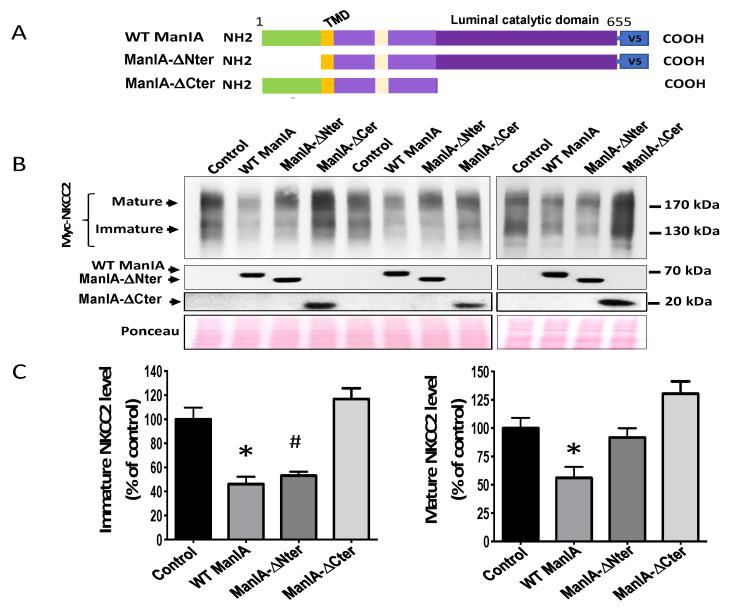
Role of the catalytic domain of ManIA on its effect on *NKCC2.* (**A**) schematic presentation of full-length WT ManIA-V5 and the truncated version of the protein devoid of its N-terminal cytoplasmic region (ManIA-ΔNter) or its luminal catalytic domain (ManIA-Cter). TMD, transmembrane domain. (**B**) Effects of WT ManIA, ManIA-Nter and ManIA-Cter on NKCC2 protein abundance. HEK cells were transiently transfected with Myc-NKCC2 either singly or in combination with WT ManIA-V5 or ManIA-ΔNter or ManIA-ΔCter. Cell lysates were subjected to SDS-PAGE and immunoblotted with anti-Myc, anti-V5 and anti-ManIA antibodies. (**C**) densitometric analysis of immature and mature NKCC2 protein levels in the absence or presence of WT ManIA or ManIA-ΔNter or ManIA-ΔCter. Data were normalized to the indicated loading control (Ponceau staining) from the same lane in each membrane and are expressed as percentage of control ±S.E. * *p* < 0.03, # *p* < 0.05 *versus* control (*n* = 4).

Previous studies reported that ManIA acts sequentially and/or in parallel with ER mannosidase and the EDEMs by trimming mannose residues of misfolded N-glycoproteins before being targeted to ERAD [57,58,59,60]. These findings prompted us to study the role of the catalytic domain of ManIA in its effect on NKCC2. To this end, we generated a construct designed ManIA-ΔCter in which we deleted the luminal catalytic domain of the enzyme (Figure 9A). The influence of this construct on the fate of NKCC2 was monitored in HEK cells under steady state conditions and was compared not only with WT ManIA but also with ManIA-Nter. In agreement with the data described in Figure 8, co-transfection with ManIA-Nter partially inhibited ManIA-induced down regulation of NKCC2 (Figure 8B,C lower left panel). Most importantly, they corroborated our observation that deleting the cytoplasmic domain of ManIA reverses its effect only on the mature form of NKCC2 (Figure 8B,C lower right panel) strongly indicating again that this region is involved in the retention of misfolded NKCC2 proteins. Remarkably, co-expression with ManIA-Cter completely abrogated the effect of the enzyme on both immature and mature NKCC2 protein expression (Figure 9B,C), clearly indicating that the catalytic domain of ManIA is required for its influence on NKCC2 total protein expression. It is worth noting that, in cells overexpressing ManIA-Cter, the effect of ManIA on NKCC2 is not only fully reversed but also the amount of NKCC2 protein abundance appeared to be even slightly higher when compared to the control group (NKCC2 alone) which, per se, may serve as an indication that ManIA-Cter may behave as a dominant negative. Altogether, these findings strongly suggest that the catalytic domain of ManIA is necessary to decrease the abundance of the cotransporter.

### 3.9. ManIA Promotes Efficient Degradation of NKCC2 Folding Mutants

**Figure 10:**
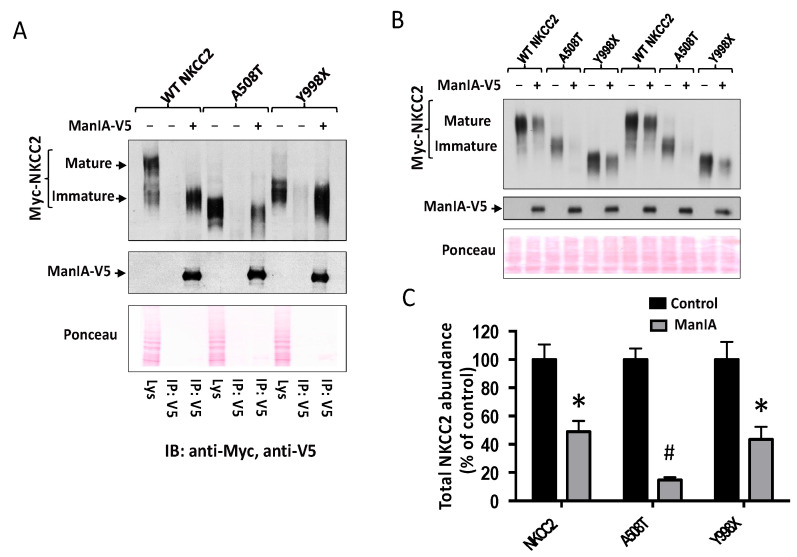
ManIA promotes the degradation of NKCC2 disease-causing mutants. (**A**) ManIA interaction with disease-causing mutants A508T and Y998X. HEK Cells were transiently transfected with Myc-NKCC2 or BS1 mutants (A508T or Y998X) either singly or in combination with WT ManIA-V5, as indicated. Cell lysates were immunoprecipitated (IP) with anti-V5 antibody. NKCC2 and ManIA proteins were detected by immunoblotting using anti-Myc and anti-V5 antibodies (**B**) ManIA effect on NKCC2 disease-causing mutants A508T and Y998X. HEK cells were transiently transfected with NKCC2 or BS1 mutants (A508T or Y998X) in the presence or absence of ManIA, as indicated. NKCC2 and ManIA proteins were detected by immunoblotting with Myc antibody and anti-V5. (**C**) Densitometric analysis of total NKCC2 proteins. *. Data are expressed as percentage of control ± S.E. *, *p* < 0.0007, #, *p* < 0.0001 (*n* = 4) versus control.

To further corroborate the notion that ManIA interferes with the cotransporter biogenesis, we next studied its influence on the fate of A508T and Y998X, two previously reported NKCC2 mutations that lead to Bartter syndrome type 1 [45,61,69]. Of note, we already provided evidence in our previous studies that A508T and Y998X mutants express only the immature and core-glycosylated form of the cotransporter [45,61]. Moreover, we proposed that Y998X mutant is retained at the ER because it is devoid of three highly conserved di-Leucine-like motifs that are required for the cotransporter exit from the ER [45,61], whereas A508T retention at the ER is very likely to be simply secondary to a folding defect. To determine whether, similar to WT NKCC2, A508T and Y998X are also substrates for ManIA-mediated degradation, we first asked if these mutants interact physically, in vivo, with the enzyme. To this end, HEK cells were co-transfected with ManIA and WT NKCC2 or its variants. As can be seen in Figure 10A, similar to WT NKCC2, the immature forms of A508T and Y998X were present in ManIA immunoprecipitates, demonstrating therefore, physical interaction between ManIA and NKCC2 mutants. Interestingly, the association of ManIA with A508T appeared to be higher than that with WT NKCC2 or Y998X, which may indicate that the folding mutant A508T is the preferred ManIA substrate, among the three proteins. Consequently, we next compared the ability of ManIA to promote the degradation of A508T and Y998X with that of WT NKCC2 by co-expressing the proteins in HEK 293 cells. As can be seen in Figure 10B,C, similar to WT NKCC2, ManIA co-expression decreased the protein level of A508T and Y998X. Most importantly while the degree of ManIA effect on Y998X was not significantly different to that seen on WT NKCC2, ManIA co-expression had clearly a more profound and severe effect on A508T (Figure 10B,C) strongly indicating that ManIA promotes the efficient ER-associated degradation of NKCC2 folding mutants.

## 4. Discussion

This study was carried out to gain insight into the molecular mechanisms underlying the regulation of the ER-associated degradation during NKCC2 biogenesis. By means of yeast two-hybrid analysis and co-immunoprecipitation assays, we identified Golgi-Mannosidase IA as a new NKCC2 binding partner. The association implicates only the immature and high-mannose glycosylated form of the cotransporter, and takes place mainly at the cis-Golgi network. We have found that ManIA knock-down increases NKCC2 protein abundance whereas its overexpression has the opposite effect. ManIA co-expression impaired NKCC2 maturation by promoting its retention, recycling to the ER and degradation via the proteasome pathway. Importantly, NKCC2 folding mutants are more prone to ManIA-mediated ERAD pathway. These findings may help to better understand how abnormalities in NKCC2 protein trafficking lead to Bartter’s syndrome which is essential for elucidating the pathophysiology of BS1 and for improving the available treatments.

It is now clearly established that mannose trimming by class I α1,2-mannosidases is involved in the recognition stage for the ERAD of glycoproteins since this process is greatly reduced by their inhibitors 1-deoxymannojirimycin and kifunensine [67,70,71,72,73]. Initially, it was thought, based on studies conducted mainly in yeast, that these compounds hamper ERAD by inhibiting ER α1,2-mannosidase I which primarily cleaves the mannose from the middle B branch of Man9 to form Man8, a glycan signal proposed to initiate ERAD [70,74]. However, in mammalian cells this hypothesis is not necessarily sound given that both kifunensine and 1-deoxymannojirimycin also inhibit Golgi α1,2-mannosidases. Furthermore, there was increasing evidence in mammalian cells demonstrating that further trimming of three to four α1,2-linked mannose residues, to form Man_6_GlcNAc_2_ (M6) or Man_5_GlcNAc_2_ (M5), contributes also to triggering the degradation of misfolded glycoproteins [32,75,76,77]. However, it was not clear which α1,2-mannosidases are responsible for this additional trimming. One candidate is ER a 1,2-mannosidase I itself since its overexpression leads to increased mannose trimming and accelerated ERAD [78,79,80]. Other possible candidates could be ER-degradation enhancing α-mannosidase-like (EDEM) proteins because both EDEM1 and EDEM3 were reported to stimulate mannose trimming and accelerate glycoprotein ERAD when overexpressed in cells [81,82]. Another possible candidate could be Golgi mannosidase IA which trims Man9 to Man6 and Man5 [31,54]. This enzyme and two other Golgi α1,2 mannosidases (*IB, and IC)* were shown to enhance the degradation and mannose trimming of an ERAD-L substrate, NHK α1-antitrypsin, when overexpressed in cultured cells [57]. In the present study, we showed evidence for a specific interaction of ManIA with NKCC2, a kidney transmembrane protein. Importantly, we demonstrated that ManIA association in vivo engages only the immature form of the cotransporter. Most importantly, we provided evidence that ManIA binding is involved in the retention and ER-associated degradation of NKCC2.

We previously provided evidence that ERAD and export from the ER constitute the major regulator of NKCC2 maturation and cell surface expression [8,44,45,46]. Indeed, we demonstrated that the majority of newly synthetized NKCC2 proteins are targeted to ER-associated degradation involving the proteasome and lysosome pathways [8,44,45,46,53]. Moreover, we showed that STCH and the protein lectin OS9 are involved in these processes by interacting with the immature form of NKCC2 mainly at the ER [46,53]. Similar to OS9 and STCH, the involvement of ManIA in the ERAD of the cotransporter was first suggested in the current study by co-immunoprecipitation assays revealing that the association of the proteins involves only the immature form of NKCC2. Of note, deleting the cytoplasmic tail of ManIA reduced but not fully inhibited its interaction with NKCC2, opening therefore the possibility for an interaction of the cotransporter with other regions of ManIA protein. Besides, despite the interaction of ManIA only with the core glycosylated and immature form of NKCC2, our immunocytochemistry studies revealed that ManIA does not co-localize with NKCC2 at the ER. With this regard, it is worth noting that ManIA was reported to be either in the Golgi or in the ER [31,83,84,85]. Another independent study reported that mannosidase IA may also reside in quality control vesicles [58]. Consequently, because the cellular distribution of ManIA may depend on the cell type, we conducted our study in two different renal cell lines, OKP and HEK cells. The localization studies performed in these cells clearly showed that ManIA is expressed in the Golgi-apparatus in both cell lines and revealed that the site of interaction with the cotransporter is the cis-Golgi network. Of note, given that the N-glycan of glycoproteins at the entrance of the Golgi apparatus is still of high mannose type, it is conceivable that NKCC2 interaction with ManIA can occur indeed at the cis-Golgi network. Using siRNA and overexpression approaches, we have shown that ManIA interacts with the immature of NKCC2 to promote its ER-associated degradation. Importantly, ManIA effect on the mature form of NKCC2 was prevented when the cytoplasmic tail of ManIA was deleted. Even more impressive, the ManIA effect on both immature and mature forms of NKCC2 was completely abrogated when the enzyme was deprived from its catalytic domain. Moreover, NKCC2 regulation by ManIA was also prevented in the presence of the proteasome inhibitor MG132, but not with chloroquine, an inhibitor of lysosomal function, indicating that ManIA targets the immature form of the cotransporter to the proteasome-dependent ERAD pathway. To further support the role of ManIA in the ERAD of NKCC2, we tested also the effect of the mannose trimming inhibitor kifunensine. Interestingly, kifunensine treatment inhibited ManIA effect on NKCC2 indicating that mannose trimming is an important process in ManIA-mediated ER-associated degradation of NKCC2. However, ManIA effect was not fully prevented by kifunensine, which implies that, at least in part, ManIA action on the cotransporter is not fully N-glycan dependent. In support of this notion, mutations of NKCC2 glycosylation sites did not fully inhibit ManIA effect on the cotransporter confirming therefore that, at least under our experimental conditions, part of the ManIA effect can be N-Glycan independent. In support of this notion, Ron et al. provided evidence that under ER stress conditions, enhanced expression of EDEM1, another alpha mannosidase protein, bypasses the mannose-trimming event and delivers the glycoprotein directly to late ERAD stages [86]. Indeed, they clearly showed that, upon overexpression of EDEM1, the degradation of the glycoprotein H2a was not blocked by kifunensine [86]. This is of particular interest because our experimental setting i.e., heterologous overexpression of secretory and membrane proteins (NKCC2) causes ER stress [87,88,89] which can explain, at least in part, the persistence of an effect of ManIA, independently of mannose trimming, on the cotransporter under these conditions. It is worth noting that the remaining ManIA effect on unglycosylated NKCC2 protein was fully blocked by MG132 further corroborating therefore the notion that ManIA promotes the ERAD of NKCC2 in a proteasome dependent pathway.

The accumulation of misfolded and aggregation-prone polypeptides is harmful to cellular health [90,91,92]. In fact, a larger number (more than 70) of human diseases result from defects in the folding of secretory and membrane proteins [91,92]. To prevent protein misfolding and maintain protein homeostasis in the secretory pathway, eukaryotes cells possess multiple quality control (QC) mechanisms [26]. They include ER quality control (ERQC) via the endoplasmic reticulum–associated degradation (ERAD) pathway [36,48] and ER-phagy [93] Golgi quality control (QCG) [38] and plasma membrane (PM) quality control [94]. Hence, misfolded proteins, in particular transmembrane proteins with complex topologies such as NKCC2, are stringently inspected by successive quality control checkpoints before reaching their final destinations [26,94]. Moreover, QC pathways can cooperate to efficiently counteract the misfolding of a single protein. For instance, although most misfolded transmembrane proteins are degraded by ERAD, some aberrant proteins, in particular under ER stress conditions, can escape ER surveillance to be packaged into COPII vesicles for anterograde trafficking to the Golgi [95,96,97]. Evidence obtained from several studies support the notion that the Golgi apparatus serves as a QC checkpoint [26,38] Indeed, misfolded or unassembled proteins that evade ERAD and ER-phagy, exit the ER and become GQC substrates that are routed to the vacuole/lysosome for degradation [26,38]. With this regard, it is very unlikely that the lysosome/vacuole-targeted GQC pathway is the mechanism underlying ManIA induced down-regulation of NKCC2, given that the lysosome inhibitor chloroquine did not prevent ManIA effect on the cotransporter. Another possibility is that some misfolded proteins entering the Golgi can be targeted for proteasomal degradation after delivery back to the ER. More precisely, the GQC captures the escaped substrates most likely in the cis-Golgi and cycles them back to the ER for ERAD by the proteasome. This is of great interest because we clearly demonstrated that ManIA colocalizes with NKCC2 mainly at the cis-Golgi network. Moreover, in contrast to chloroquine, the proteasome inhibitor MG132 abolished ManIA effect on NKCC2, strongly suggesting therefore that the proteasome-targeted GQC is very likely to be the main pathway governing NKCC2 regulation by ManIA. Of note, a very recent study in yeast proposed that some misfolded proteins entering the Golgi can be targeted directly for proteasomal degradation without being cycled back to the ER, a mechanism called EGAD [98]. However, although one cannot exclude this additional mechanism, we clearly showed that upon ManIA co-expression, NKCC2 is largely retained at the ER. Hence, our data strongly suggest ManIA promotes the retention, recycling, and the proteasome dependent ERAD of misfolded NKCC2 proteins that initially escape the ER quality control.

Exhaustive exploration of the mechanisms underlying the ERAD machinery has furnished several new insights into how ERAD contributes to human health during both normal and diseased states [48,92]. The importance of ER quality control, in general, and the ERAD phenomenon, in particular, has been mentioned in a large number of human pathologies, called conformational diseases [48,92]. In regards to NKCC2, we previously documented that Bartter syndrome type 1 is among diseases linked to the ERAD pathway [61]. Indeed, although distinct cellular pathways may account for NKCC2 loss of function in BS1 disease, our data revealed that ER-associated protein degradation is the most common mechanism underpinning BS1 [61]. Accordingly, identification of proteins that bind specifically to the immature forms of WT NKCC2 and its folding mutants is important to decipher the molecular mechanisms underlying the regulation of their ERAD. In the present study, we provided evidence that in addition to WT NKCC2, ManIA interacts with the immature form of NKCC2 mutants A508T and Y998X. Moreover, we demonstrated that ManIA co-expression has a more profound effect on NKCC2 folding mutant A508T when compared to WT NKCC2, strongly suggesting that ManIA may have an important role in the ERAD of NKCC2 mutants during BS1. This is of particular interest because we previously reported that A508T and Y998X are retained at the ER [45,61]. Given that our immunocolocalization experiments revealed that, independently of the expression system, ManIA is expressed at the cis-Golgi network, the interaction of the enzyme with these two NKCC2 mutants strongly suggest that even though most A508T and Y998X are largely trapped in the ER, there is significant transfer of these two mutants from the ER to the cis-Golgi where they can be captured by ManIA to be delivered back to the ER. With this regard, Hosokawa et al. showed also that, although most of transfected NHK proteins are retained in the ER, some of them evaded the ER quality control and to reach the cis-Golgi where they are trimmed by Golgi a 1,2-mannosidases to be targeted for ERAD [57]. Furthermore, several recent studies demonstrated that ERManI, which was initially predicted to function in the ER, was also localized to the Golgi complex in mammalian cells, where it contributes to a Golgi-based quality control checkpoint that facilitates the retrieval of captured ERAD substrates back to the ER [59,60,99]. Of note, given that, like NKCC2, ManIA is transmembrane protein, it may also transiently interact with the cotransporter at the ER allowing therefore the enzyme to participate in generating the signal that targets misfolded NKCC2 proteins for ERAD either as they transit through the ER or on their way to the Golgi.

In summary, we found that Golgi ManIA interacts with the immature form of NKCC2 at the cis-Golgi to promote its ER retention and to accelerate its ERAD by the proteasome pathway. To the best of our knowledge, this is the first study describing a major role of the Golgi quality control mechanism in NKCC2 biogenesis. Moreover, this also is the first report providing evidence that besides luminal proteins, ManIA can be also involved in the ERAD of transmembrane proteins. Our data strongly suggest that ManIA maneuvers within a cis-Golgi-localized quality control checkpoint to serve as a backup system to ERAD surveillance in the ER, providing therefore a powerful additional network for adequate removal of unwanted misfolded NKCC2 proteins protecting therefore cells from proteotoxicity caused by the formation of protein aggregates, in particular during Bartter syndrome disease. Undeniably, ManIA cannot work in isolation to mediate the ERAD of the cotransporter. Appropriately, one may plausibly postulate that ManIA works in concert, sequentially or simultaneously, with other NKCC2 binding-proteins and ERAD components, such as OS9 [46] and STCH [53] to mediate the ER quality control of the cotransporter. With this regard, we propose a model whereby, under ER stress conditions: (1) OS9 is involved in the retention of misfolded NKCC2 proteins at the ER and its ERAD by the proteasome. (2) STCH plays a crucial role in the ERAD of NKCC2 by the proteasome and lysosome pathways. (3) Golgi ManIA contributes in the ERAD of NKCC2 by capturing misfolded NKCC2 proteins that escaped ER quality control and delivers them back to the ER. Obviously, further experiments are needed to uncover the precise mechanism behind this model. The thorough characterization and identification of the specific molecular component of the ER and Golgi quality controls of NKCC2 and its disease-causing mutants may offer a foundation to define new therapeutic strategies targeting cotransporter transport from the ER and Golgi networks to the cell surface.

## Data Availability

The data that support the findings of this study are available on request from the corresponding author.
